# Different MicroRNA Profiles in Chronic Epilepsy Versus Acute Seizure Mouse Models

**DOI:** 10.1007/s12031-014-0368-6

**Published:** 2014-07-31

**Authors:** Anita Kretschmann, Benedicte Danis, Lidija Andonovic, Khalid Abnaof, Marijke van Rikxoort, Franziska Siegel, Manuela Mazzuferi, Patrice Godard, Etienne Hanon, Holger Fröhlich, Rafal M. Kaminski, Patrik Foerch, Alexander Pfeifer

**Affiliations:** 1 Institute of Pharmacology and Toxicology, University of Bonn, Sigmund-Freud-Str. 25, 53127 Bonn, Germany; 2 UCB Pharma, Chemin du Foriest, 1420 Braine-l’Alleud, Belgium; 3 Bonn-Aachen International Center for Information Technology (B-IT), Algorithmic Bioinformatics, University of Bonn, Dahlmannstr. 2, 53113 Bonn, Germany; 4 IP & Science, Thomson Reuters, 5901 Priestly Dr., #200, Carlsbad, CA 92008 USA; 5 PharmaCenter, University of Bonn, 53127 Bonn, Germany

**Keywords:** Epilepsy, miRNA, Status epilepticus, Microarray, Hippocampus

## Abstract

Epilepsy affects around 50 million people worldwide, and in about 65 % of patients, the etiology of disease is unknown. MicroRNAs are small non-coding RNAs that have been suggested to play a role in the pathophysiology of epilepsy. Here, we compared microRNA expression patterns in the hippocampus using two chronic models of epilepsy characterised by recurrent spontaneous seizures (pilocarpine and self-sustained status epilepticus (SSSE)) and an acute 6-Hz seizure model. The vast majority of microRNAs deregulated in the acute model exhibited increased expression with 146 microRNAs up-regulated within 6 h after a single seizure. In contrast, in the chronic models, the number of up-regulated microRNAs was similar to the number of down-regulated microRNAs. Three microRNAs—miR-142-5p, miR-331-3p and miR-30a-5p—were commonly deregulated in all three models. However, there is a clear overlap of differentially expressed microRNAs within the chronic models with 36 and 15 microRNAs co-regulated at 24 h and at 28 days following status epilepticus, respectively. Pathway analysis revealed that the altered microRNAs are associated with inflammation, innate immunity and cell cycle regulation. Taken together, the identified microRNAs and the pathways they modulate might represent candidates for novel molecular approaches for the treatment of patients with epilepsy.

**Electronic supplementary material:**

The online version of this article (doi:10.1007/s12031-014-0368-6) contains supplementary material, which is available to authorized users.

## Introduction

According to the World Health organisation and the International Bureau for Epilepsy (IBE), epilepsy is a chronic neurologic disorder that affects 4 to 10 per 1,000 people, with 20–30 % of them experiencing more than one seizure per month (Forsgren et al. 2005). The most common type of focal epilepsy is temporal lobe epilepsy (TLE) characterised by spontaneous recurrent seizures (SRSs) (Weiss et al. 1986), neuronal loss in the hippocampus and mossy fibre sprouting (Riban et al. 2002). A significant proportion of patients with TLE are resistant to antiepileptic drugs (AEDs), which is in stark contrast with the high efficacy of the same AEDs observed in acute preclinical models of seizures (Loscher and Schmidt 2011). AEDs not only fail to generate seizure freedom but are also ineffective in modifying the process of epileptogenesis especially in high-risk patients (Benardo 2003; Temkin 2009; Loscher and Schmidt 2011). Molecular profiling studies have provided insight into the molecular changes that contribute to the epileptogenic process, as well as potential mechanisms, ranging from transcription factors (McClelland et al. 2011; Mazzuferi et al. 2013) to chromatin methylation (Kobow and Blumcke 2011) and to small non-coding RNAs (Jimenez-Mateos et al. 2011, 2012; McKiernan et al. 2012).

MicroRNAs (miRNAs) are small non-coding RNAs involved in post-transcriptional regulation of gene expression (Aravin and Tuschl 2005; Pfeifer and Lehmann 2010). miRNAs are transcribed by RNA polymerase II; they form a stem-loop structure that is further processed by a microprocessor complex (including the enzyme Drosha) into precursor miRNA (Gregory et al. 2006) and cleaved by RNase III enzyme *Dicer* into 21–22-nucleotide (nt) long miRNA duplexes. One strand of the duplex is loaded onto the RNA-inducing silencing complex (RISC) as mature miRNA that binds to its complementary sequences in the 3′ untranslated region (3′ UTR) of target messenger RNAs (mRNAs). Even partial complementary binding of the miRNA to the 3′ UTR of the target mRNA can result in post-transcriptional inhibition. Therefore, one miRNA can regulate potentially several hundred transcripts (Hutvagner and Zamore 2002; Pillai 2005).

miRNAs have been identified across species and are thought to play essential roles in a variety of fundamental cellular processes from cell death to neurogenesis (Volvert et al. 2012). miRNA numbers increase with the complexity of the organism suggesting that miRNAs are contributing to an additional layer of plasticity and a more sophisticated regulation of gene expression in higher organisms (Griffiths-Jones et al. 2006; Lee et al. 2007). Interestingly, miRNAs are generally more highly abundant in the brain relative to other tissues (Kosik 2006). Ablation of *Dicer* in the mouse brain blocks miRNA biogenesis leading to neuronal loss and premature death indicating that miRNAs are important for brain function (Schaefer et al. 2007). Several miRNAs regulate brain development, cellular function and neurological diseases (Im and Kenny 2012; McNeill and Van Vactor 2012; Salta and De Strooper 2012). Furthermore, recent reports identified individual miRNAs that influence seizure-induced neuronal death (Jimenez-Mateos et al. 2011, 2012; McKiernan et al. 2012). miRNA profiling studies in epileptic tissues have revealed highly selective, spatiotemporal changes in expression patterns; however, inconsistencies between the deregulated miRNAs have been observed. This is likely due to differences in species (rat versus mouse) and models used (chemically versus electrically induced status epilepticus (SE)). In addition, different time points after SE as well as miRNA profiling techniques were applied. Therefore, additional miRNA profiling studies are needed to allow a more comprehensive characterisation of miRNA involvement in epilepsy pathophysiology (Jimenez-Mateos and Henshall 2013).

Several rodent models of human TLE have been developed (Turski et al. 1983; Mazzuferi et al. 2012). In these models, a chemical or electrical stimulus induces SE after which the animals develop spontaneous recurrent epileptic seizures (SRS), a hallmark of chronic epilepsy. These animals display signs of hippocampal reorganisation, neuronal loss and neuroinflammation with some similarity to different stages of the human disease. In addition, seizures emanating from temporal lobe structures can be elicited by electrical stimulation in naïve animals, which otherwise do not display any TLE-related pathology. One important example of such an approach is the 6-Hz seizure model, in which naïve mice display brief focal (limbic) convulsions after acute electrical stimulation (Barton et al. 2001). As such, there is a fundamental difference between post-SE models, where the animals display SRS (epilepsy models) and models where a single seizure is acutely induced (seizure models).

For the present study, we took advantage of the differences between epilepsy and seizure models to delineate common changes in miRNA expression profiles observed in chronic epilepsy models (pilocarpine and self-sustained status epilepticus (SSSE)) and after induction of an acute seizure (6-Hz model). We observed limited overlap between deregulated miRNAs in the acute seizure model and the chronic models. However, despite the different stimuli used for induction of SE (chemical versus electrical), there was a significant overlap in altered miRNA patterns between the two chronic models. Pathway analysis revealed a potential involvement of inflammation, innate immunity and cell cycle regulation which could lead to novel therapeutic approaches in epilepsy.

## Material and Methods

### Ethics Statement

All procedures were carried out according to the Helsinki declaration and conducted according to the guidelines of the European Community Council directive 86/609/EEC. The protocol (reference: PILO-STATUS-EPILEPTICUS-MO, ELEC-6HZ-SEIZURE-MO, STIM-ELEC-STATUS-EPILEPTICUS-MO) was approved by UCB’s Ethical Committee for Animal Experimentation (accreditation number from Service Public Fédéral Santé publique: LA 122 0040).

### Study Design

We compared hippocampal miRNA expression patterns in two mouse models of chronic epilepsy (pilocarpine and SSSE) and in an acute seizure model (6 Hz). The expression of 579 miRNAs was analysed using a miRNA microarray profiling platform. An outline of the study is depicted in Supplementary Fig. 1. In both chronic epilepsy models, animals developed SRS following induction of SE, and samples were taken at an early time point (i.e. 24 h after induction of SE) and at a late time point (i.e. 28 days after induction of SE). In the acute seizure model, animals displayed an acute, single focal seizure, and hippocampal tissue was dissected at 3, 6, 24 and 72 h following the acute insult. A detailed bioinformatics analysis was performed including principal component analysis (PCA), heat map analysis as well as Venn diagrams for the analysis of miRNA expression pattern across all three models at different time points. Significantly deregulated miRNAs (*p* < 0.05) were validated by quantitative reverse transcription polymerase chain reaction (RT-qPCR).

### Animals

For the chronic epilepsy models, 5–6-week-old male mice (Charles River) were subjected to two different models of chronic epilepsy causing SRSs, i.e. pilocarpine and SSSE models. Selection of an appropriate mouse strain for particular epilepsy models is critical due to well documented differences in seizure vulnerability among various mouse strains (Schauwecker 2011). Therefore, both pilocarpine and SSSE models had been previously established and optimised in our lab using two different mouse strains, Naval Medical ResearchInstitute (NMRI) and C57Bl/6 J, respectively (Niespodziany et al. 2010; Mazzuferi et al. 2012).

A single injection of pilocarpine was used to trigger SE in male NMRI mice (28–32 g at the beginning of the study). As previously described (Mazzuferi et al. 2012), animals were injected intraperitoneally (i.p.) with 1 mg/kg of N-methylscopolamine bromide 30 min prior to pilocarpine treatment (300 mg/kg; i.p.). Within 10 to 45 min after pilocarpine treatment, animals displayed generalised clonic-tonic seizures that progressed to continuous convulsive activity, i.e. SE. The duration of SE lasted up to 2 h and was interrupted by i.p. injection of diazepam (10 mg/kg) to limit the extent of brain damage. The mice surviving SE typically show SRSs within few days and continue to display them for several weeks (Mazzuferi et al. 2012). Therefore, the mice were scarified 24 h after SE induction (early time point) without monitoring for SRS, while all mice were continuously video monitored for 28 days and scarified to collect brain samples (late time point) (Supplementary Figs. 2 and 3). Naïve, age-matched mice of the same strain were used as controls for each time point.

SSSE is a chronic epilepsy model induced by electrical stimulation. As previously described (Niespodziany et al. 2010), C57Bl/6 J male mice were surgically implanted with electroencephalography (EEG) electrodes: depth electrode (bipolar) AP = −1.40 mm, *L* = −2.65 mm, *D* = −5.00 mm; cortical electrode (monopolar) AP = −4.00 mm, *L* = +3 mm and reference electrode in the prefrontal bone. After recovery from surgery, mice underwent electrical stimulation through the amygdala-implanted electrode (90-min duration, 100-ms trains of 1-ms alternating current pulses (50 Hz), 2 trains per 1 s, 250-μA peak current intensity). Upon cessation of electrical stimulation, the animals developed SSSE represented as continuous convulsive activity that was stopped after 150 min by i.p. injection of diazepam (10 mg/kg). SRSs typically start to occur within a few days after SSSE (Niespodziany et al. 2010). By analogy to the pilocarpine model, the mice were scarified 24 h after SSSE induction (early time point) without monitoring for SRS, while all mice were continuously video-EEG monitored for 28 days and scarified to collect brain samples (late time point) (Supplementary Figs. 2 and 3). Naïve, age-matched mice of the same strain were used as controls for each time point. For both the pilocarpine and the SSSE model, eight animals were used per experimental group.

For acute temporal lobe seizures, male NMRI mice were used. Mice were electrically stimulated to induce a single seizure activating only temporal lobe structures (Barton et al. 2001). As previously described (Kaminski et al. 2004), seizures were triggered by a stimulator (ECT Unit 57800, Ugo Basile, Comerio, Italy) using a current intensity of 44 mA, 0.2-ms monopolar pulses at 6-Hz frequency for a duration of 3 s through corneal electrodes. Prior to stimulation, a drop of saline with 0.1 % Unicaïne was placed on the eyes to ensure good conductivity and mild anesthesia. After stimulation, each mouse was observed for convulsive behaviour (i.e. stereotypy, immobility and mild myoclonus), typically lasting more than 7 s. As controls age-matched, non-stimulated animals were used. In this model, seven animals per experimental group were used.

A modified Racine scale (Racine 1972) was used to characterise convulsive motor seizure severity as follows: Stage 3 seizures were defined by forelimb clonus and a lordotic posture, stage 4 seizures included forelimb clonus and rearing and stage 5 seizures displayed a stage 4 seizure with loss of the righting reflex (Williams et al. 2009). For all models, the naive control animals underwent similar procedures as the treated animals with the exception of induction of seizure or SE.

### Tissue Collection and RNA Isolation

Mice were sacrificed 24 h and 28 days after the induction of SE in both, pilocarpine and SSSE model. As control groups, 24-h and 28-day time points were used for the pilocarpine model, and a 28-day time point was used for the SSSE model for technical reasons. Mice were sacrificed at 3, 6, 24 and 72 h following acute seizure in the 6-Hz model. Mouse hippocampi were extracted and snap frozen. Total RNA was isolated from the sonicated tissue using miRVana miRNA isolation kit (Ambion Inc., Austin, Texas, USA), and RNA quality was verified (Agilent Bioanalyzer Lab-on-a-Chip System) observing intact 5S, 5.8S and 18S ribosomal RNA. All samples analysed had an RNA integrity number (RIN) >7.

### MicroRNA Microarray

Profiling was performed using the miRCURY^tm^ LNA Array miRNA on dual-channel arrays (Exiqon A/S) with complete coverage of miRBase release 14. For pilocarpine and SSSE models, miRNA microarray profiling was performed at 24 h and 28 days following SE. For 6-Hz model, miRNA microarray profiling was done at 3, 6, 24 and 72 h after acute seizure. Based on comparison with the current miRBase® release 20 (Griffiths-Jones 2006; Griffiths-Jones et al. 2008), all sequences not representing miRNAs were deleted from the analysis.

### Normalisation and Preprocessing

Data were preprocessed and analysed using bioconductor® (Gentleman et al. 2004) and R® (R Development Core Team 2011) tools. Red and green intensities and their respective background values were extracted from two-channel arrays. In order to avoid negative corrected intensities and to reduce variability of low intensity log-ratios, the norm exponential convolution method was used for background correction (Smyth 2005; Ritchie et al. 2007; Silver et al. 2009). Background-corrected values were then normalised and summarised to average log intensities and intensity log-ratios using loess normalisation for within-array normalisation and quantile normalisation for between-array normalisation, considering spot quality weights (Yang et al. 2002; Smyth and Speed 2003; Rao et al. 2008; Risso et al. 2009). A rigorous quality assessment confirmed the quality of the chips. The GAL file from Exiqon together with the 20th release of miRBase® (Griffiths-Jones 2004; Kozomara and Griffiths-Jones 2011) was used for chip annotation. Data sets were submitted to Gene Expression Omnibus (GEO) with the following accession numbers: GSE51840 (6-Hz data set), GSE51841 (pilocarpine data set) and GSE51842 (SSSE data set).

### Differential Expression Analysis

Differential expression analyses for miRNAs were performed using “limma” linear models for microarray data (Smyth 2005) utilising the empirical Bayes method (Casella 1985). Statistical dependencies of samples between different conditions and replicates were considered via a factorial design matrix in limma using a “condition-replicate” factor. Contrasts were considered for interaction effects. Correlations between the technical quadruplicates in the chips were taken into consideration, and spot quality weights were used (manually flagged spots, empty, poor and negative spots are down-weighted with 0.7, 0.4, 0.2 and 0.1 factors, respectively). Corrections for multiple testing were done using Benjamini and Hochberg’s method (Benjamini and Hochberg 1995). Significant differentially expressed miRNAs were reported at FDRBH < 0.05. In the differential expression analyses, we compared pilocarpine-treated mice samples to their counterpart naive samples at 24 h and 28 days, respectively. SSSE samples at 24 h and 28 days were compared to a single SSSE control group. In 6-Hz data, samples at the subsequent time points (3, 6, 24 and 72 h) were compared to the samples at the initial time point (0 h).

### RT-qPCR

Universal complementary DNA (cDNA) synthesis kits, SYBR Green master mix and miRCURY LNA Universal RT murine miRNA PCR primer sets (Exiqon A/S) were used for quantification of 135b-5p, miR-142-3p, miR-142-5p, miR-34b-3p, miR-140-5p, miR-212, miR-221, miR-222 and miR-2137. Synthesis of cDNA was performed according to Exiqon standard protocols using 20 ng of total RNA. RNA and cDNA concentrations were measured by Nanodrop 2000c Spectrophotometer (Peqlab Biotechnology). Real-time PCR reactions were carried out in 384 well plates with 5-μl SYBR Green master mix, 1 μl of primer mix for each miRNA and 4 μl of 1:80 diluted cDNA per well. Each sample was run in triplicates for the miRNA of interest as well as for endogenous controls (RNU6). The real-time PCR assays were performed on a 7900HT system (Life Technologies). The real-time PCR settings were 50 °C for 2 min, 95 °C for 10 min, 40 cycles of 95 °C for 10 s followed by 60 °C for 1 min and 25 °C for 1 min. Data were calculated using the ΔΔCt method (Livak and Schmittgen 2001).

### Pathway Analysis

Pathway analysis was conducted with MetaCore™ software suite (http://thomsonreuters.com/site/systems-biology). This software employs a dense and manually curated database of interactions between biological objects and variety of tools for functional analysis of high-throughput data including pathway map enrichment (Nikolsky et al. 2005).

First, for all the 728 canonical pathway maps available in the database, miRNAs binding to the corresponding genes were identified. Only interactions with a high level of confidence were taken into account (proven interaction in at least one animal model). Then, an enrichment analysis was performed comparing the list of differentially expressed miRNAs with the list of miRNAs associated to each pathway map. The enrichment analysis was performed using a hyper-geometric test separately for up- and down-regulated miRNAs. A correction for multiple testing was done using Benjamini and Hochberg’s method (Benjamini and Hochberg 1995), and significant canonical pathway maps were selected according to a FDR < 0.05.

## Results

### Relative Comparison of Chronic Epilepsy and Acute Seizure Models

**Fig. 1:**
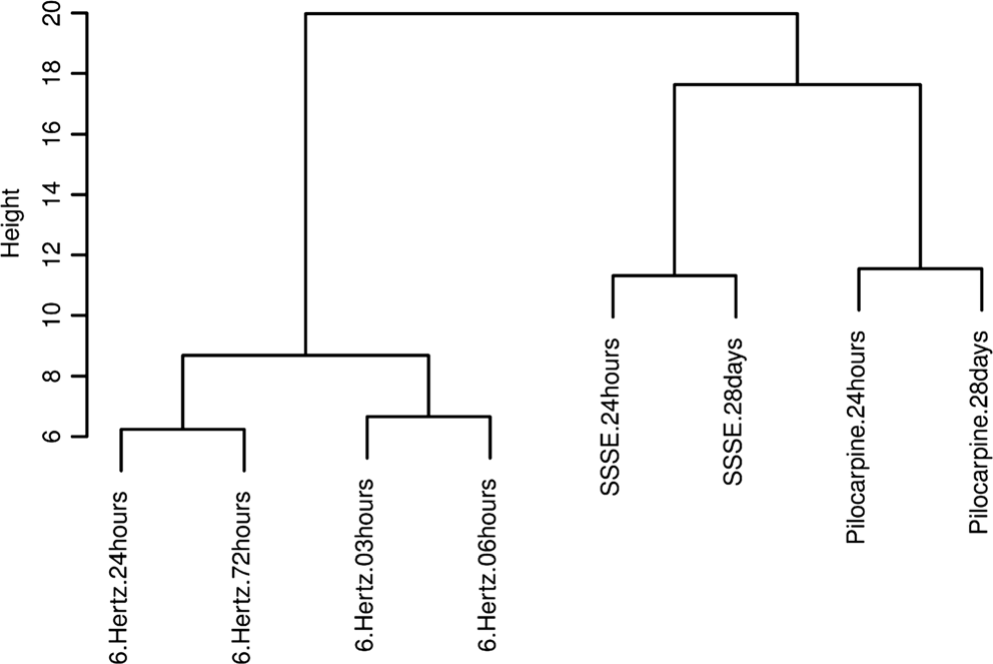
Clustering of miRNAs in different epilepsy mouse models. Hierarchical clustering of miRNAs extracted from mouse hippocampus of the pilocarpine and SSSE models 24 h and 28 days following SE, as well as in the 6-Hz model 3, 6, 24 and 72 h following seizure

To analyse whether the two chronic epilepsy models (pilocarpine, SSSE) and the acute seizure model (6 Hz) have distinct miRNA expression profiles, we performed a complete linkage hierarchical clustering using Euclidean distance as similarity measure (Fig. 1). This clustering was based on all miRNAs detected in the expression profiling of the pilocarpine and SSSE models at the time points 24 h and 28 days as well as of the 6-Hz model at the time points 3, 6, 24 and 72 h.

The dendrogram (Fig. 1) showed a clear segregation of the acute seizure and chronic epilepsy models implying a closer correlation of the miRNA pattern for the two chronic models than with the acute seizure model. Also, the PCA of all three models revealed a clustering of the two epilepsy models versus the seizure model, in accordance with the dendrogram (Supplementary Fig. 4). Furthermore, for the chronic models, different time points within a model clustered closer relative to the same time point in the other SE animals indicating some model-specific changes in the miRNA expression pattern. In the 6-Hz model, miRNA expression clustered more closely at early time points (3 and 6 h) relative to the later time points (24 and 72 h) (Fig. 1). The most prominent difference was observed between the first (3 h) and the last time point at 72 h. Thus, similar to the chronic model also in the acute model, subsets of miRNAs were differentially regulated at early and late time points.

### Principal Component Analysis in Chronic Epilepsy and Acute Seizure Models

**Fig. 2:**
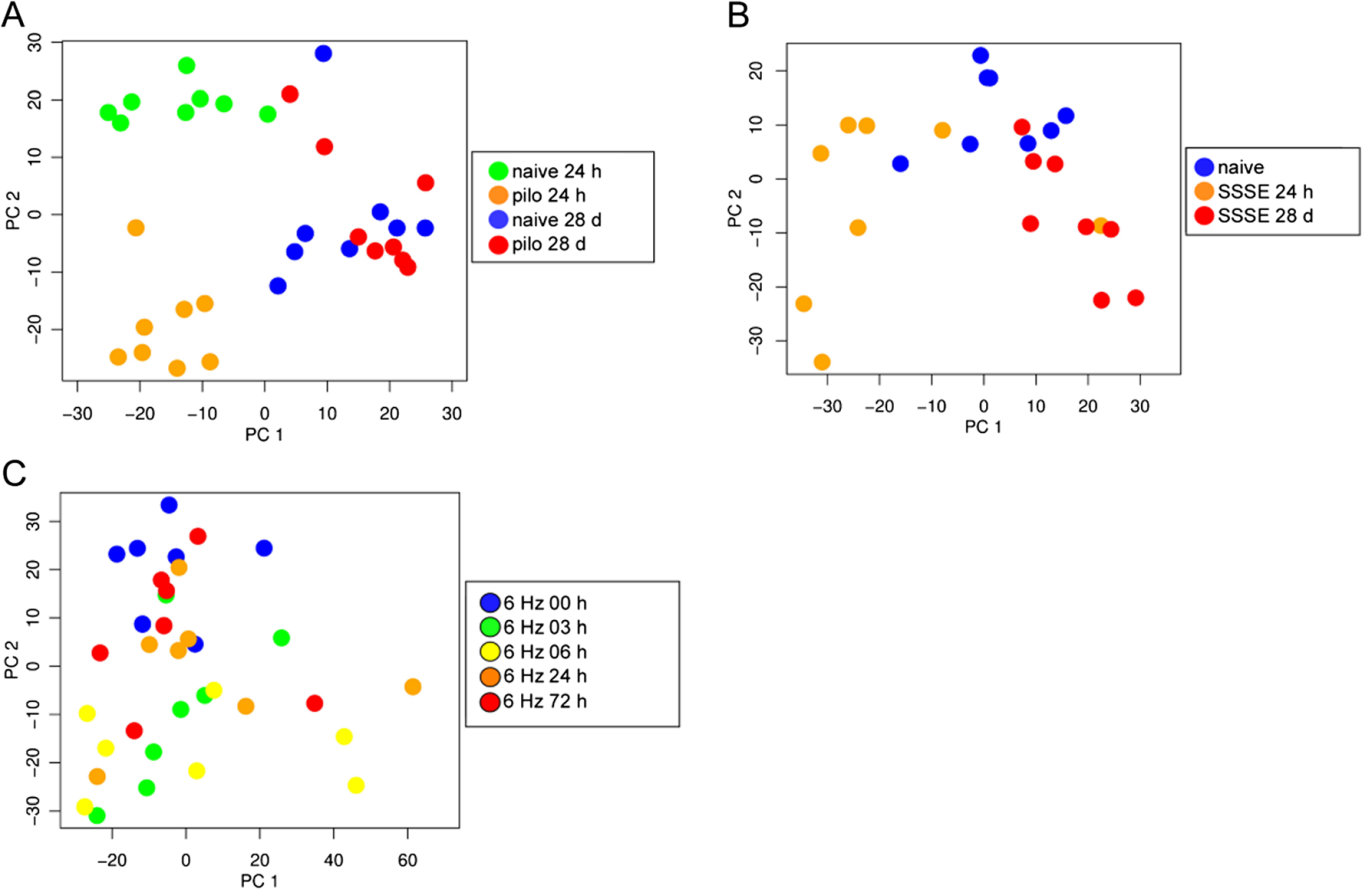
Principal component analysis (PCA) based on all detected miRNAs. Scores of the samples in the first and the second principal components are shown. Clustering of samples of **a** animals of the pilocarpine model: 24 h and 28 days naïve versus 24 h and 28 days after pilocarpine-induced status epilepticus (SE); **b** animals of the SSSE model: 24 h and 28 days naïve versus 24 h and 28 days following electrically induced SE; **c** animals of the 6-Hz model: 0 (naïve), 3, 6, 24 and 72 h following seizure induction

A PCA was performed in order to assess the relationship between samples according to the miRNA expression profiles (Fig. 2). The PCA was performed for each model separately including all detected miRNAs. In the pilocarpine model, the early time point (24 h) and the naïve 24 h formed clearly separate clusters, while at the later time point (28 days), clustering is less obvious (Fig. 2a). In the SSSE model, the different groups clustered together with some overlaps including one outlier for the early time point (24 h) (Fig. 2b). In the 6-Hz model, the grouping of the samples was less clear. Nevertheless, the expression of miRNAs in the epileptic animals is more different to naïve animals 3 and 6 h after a single seizure as compared to 24 and 72 h (Fig. 2c).

### miRNA Expression Pattern in Pilocarpine, SSSE and 6-Hz Models

**Fig. 3:**
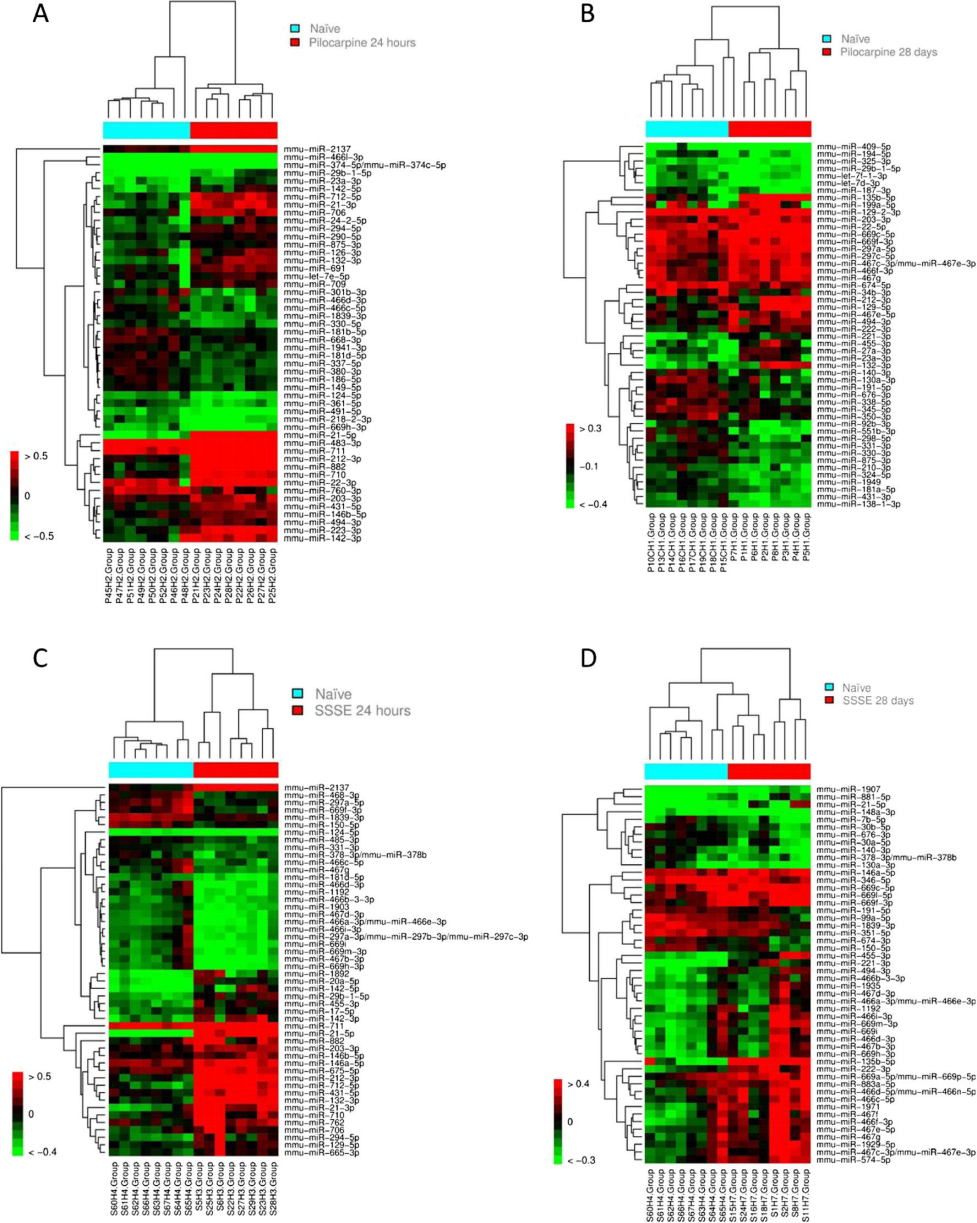
Unsupervised hierarchical clustering analysis for the expression values of the differentially expressed miRNAs. miRNAs with FDR_BH_ < 0.05 are shown. The *red* and *green squares* represent the up- and down-regulated miRNAs, respectively. Animals from the control groups (naïve) are labelled in *turquoise* and TLE animals are labelled in *red*. Heat map showing miRNA expression in naïve mice (0 h) versus induced mice (**a**–**d**). **a** pilocarpine-induced SE mice (24 h) (up-regulated miRNAs ratio > 0.5, down-regulated miRNAs ratio < −0.5). **b** Pilocarpine-induced SE mice (28 days) (up-regulated miRNAs ratio >0.3, down-regulated miRNAs ratio < −0.4). **c** Electrically induced SE mice (24 h) (up-regulated miRNAs ratio >0.5, down-regulated miRNAs ratio < −0.4). **d** Electrically induced SE mice (28 days) (up-regulated miRNAs ratio >0.4, down-regulated miRNAs ratio < −0.3) (colour figure online)

Next, we performed a two-way hierarchical clustering using the top 50 significantly deregulated miRNAs showing a *p* value <0.05 (Benjamini and Hochberg corrected) for each model analysed separately per time point (Fig. 3).

In the pilocarpine model, especially the early time point (24 h) showed a clear separation compared with naïve animals (Fig. 3a), while at the later time point (28 days), clustering and group separation are less pronounced (Fig. 3b). The number of deregulated miRNAs at 24 h (99) is higher relative to the 28-day samples, where expression of 51 miRNAs was significantly altered (Supplementary Table 1). In the SSSE model, we observed a clear clustering of naïve and epileptic animals at 24 h (Fig. 3c). However, a less pronounced clustering was observed in SSSE animals at 28 days (Fig. 3d).

**Fig. 4:**
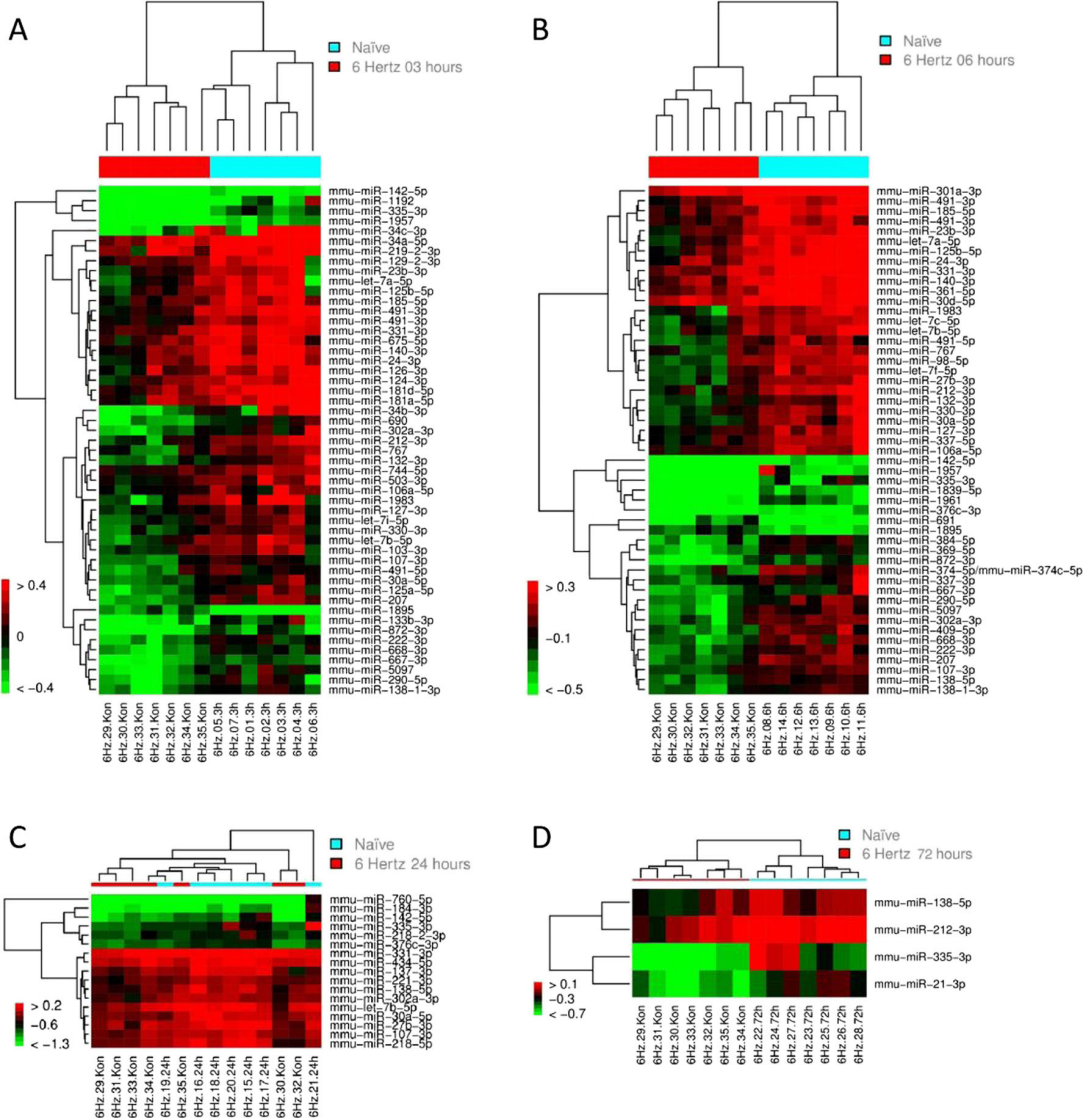
Unsupervised hierarchical clustering analysis for the expression values of the differentially expressed miRNAs. miRNAs with FDR_BH_ < 0.05 are shown. Colour labelling for up- and down-regulated miRNAs as well as for different animal groups as in Fig. 3. **a** Heat map showing miRNA expression in naïve mice (0 h) versus single seizure mice (3 h) (up-regulated miRNAs ratio >0.4, down-regulated miRNAs ratio < −0.4). **b** Heat map showing miRNA expression in naïve mice (0 h) versus single seizure mice (6 h) (up-regulated miRNAs ratio >0.3, down-regulated miRNAs ratio < −0.5). **c** Heat map showing miRNA expression in naïve mice (0 h) versus single seizure mice (24 h) (up-regulated miRNAs ratio >0.2, down-regulated miRNAs ratio < −1.3). **d** Heat map showing miRNA expression in naïve mice (0 h) versus single seizure mice (72 h) (up-regulated miRNAs ratio >0.1, down-regulated miRNAs ratio < −0.7)

Whereas in the chronic models, a clear clustering was observed for the majority of time points; in the 6-Hz model, this effect was less pronounced at 3 and 6 h after seizure (Fig. 4a, b). At 24 and 72 h after SE, segregation among groups was even less evident (Fig. 4c, d). In addition, the total number of miRNAs with an altered expression level was significantly reduced at the later period (24 and 72 h) compared to time points immediately after a seizure (3 and 6 h).

### Overlapping miRNA Expression Profiles in Seizure Models

**Fig. 5:**
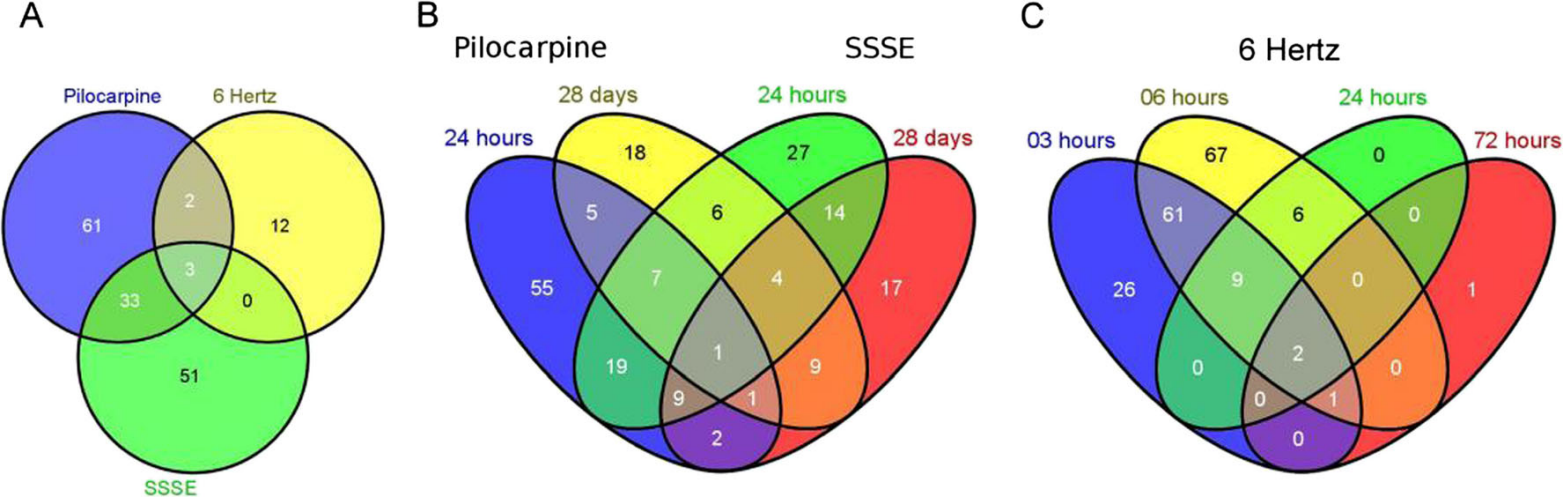
Venn diagram of all significantly deregulated miRNAs (FDR_BH_ < 0.05) in the different models. **a** Venn diagram showing the overlap of commonly deregulated miRNAs in 6 Hz, pilocarpine and SSSE model at 24 h following induction of seizure or SE. **b** Venn diagram showing expression of deregulated miRNAs in the chronic pilocarpine and the SSSE model at 24 h and 28 days following SE. **c** Venn diagram showing deregulated miRNAs in the acute 6-Hz model at 3, 6, 24 and 72 h following single seizure

To identify miRNAs potentially associated with chronic forms of epilepsy, we investigated the overlap of miRNA expression between the two chronic epilepsy models (pilocarpine and SSSE) and the acute seizure model (6 Hz) using Venn diagrams (Fig. 5). Again, the analysis was based on all differentially expressed miRNAs (*p* value <0.05) between epileptic and control animals within each model.

First, the data for the 24-h time point were compared among the three models, as this time point is common to all three models. The Venn diagram showed an overlap of three miRNAs between the acute and chronic models at the 24-h time point (Fig. 5a). Although the three miRNAs (miR-142-5p, miR-331-3p and miR-30a-5p) were differentially regulated, only miR-142-5p was consistently up-regulated in all three models. In contrast, miR-331-3p and miR-30a-5p were up-regulated in the chronic models and down-regulated in the 6-Hz model at 24 h. Two miRNAs were specifically overlapping between the pilocarpine and the 6-Hz model; miR-335-3p was consistently up-regulated, while miR-218-2-3 showed a different regulation in both models (Fig. 5a). There was no overlap between the two electrically stimulated models at 24 h (Fig. 5a). Taken together, the number of overlapping miRNAs between the acute and chronic models was lower compared to the corresponding miRNAs (33) between the two chronic models.

Furthermore, a detailed comparison of the chronic models is shown in Fig. 5b. Among all significantly deregulated miRNAs in both chronic models, only miR-494-3p was consistently up-regulated under all experimental conditions. The number of commonly deregulated miRNAs was higher (36 miRNAs) at the early (24 h) versus the late (28 days) time point (15 miRNAs, including miR-676, miR-467c/miR-467e, miR-191, miR-669c and miR-181). The annotations for these miRNAs are shown in the Supplementary Table 1. Thirty-six miRNAs deregulated at the acute time point (24 h) represent 36.4 and 41.4 % of all the deregulated miRNAs in the pilocarpine and SSSE model, respectively. Similarly, 15 miRNAs at the late time point (28 days) represent 29.4 and 26.3 % of all deregulated miRNAs in the pilocarpine and SSSE model, respectively. These results indicate a larger overlap of the models at the early time point compared to the later stage of the model.

Analysis of the different time points in the acute seizure model (6 Hz) revealed that only early time points (3 and 6 h) displayed substantially more deregulated miRNAs, while at later time points (24 and 72 h), only few miRNAs were deregulated (Fig. 5c). The majority of miRNAs (146) were significantly deregulated at the 6-h time point. Seventy-three miRNAs were identified as overlapping at the earliest time points representing 73.7 and 50 % of miRNAs detected at 3 and 6 h, respectively. Of note, two miRNAs were commonly deregulated across all time points of the 6-Hz model: miR-335-3p and miR-138-5p. Taken together, our data show that the majority of changes in differential miRNA expression were detected within 24 h following the initial insult in the acute seizure model.

### Up- and Down-Regulated miRNAs

**Fig. 6:**
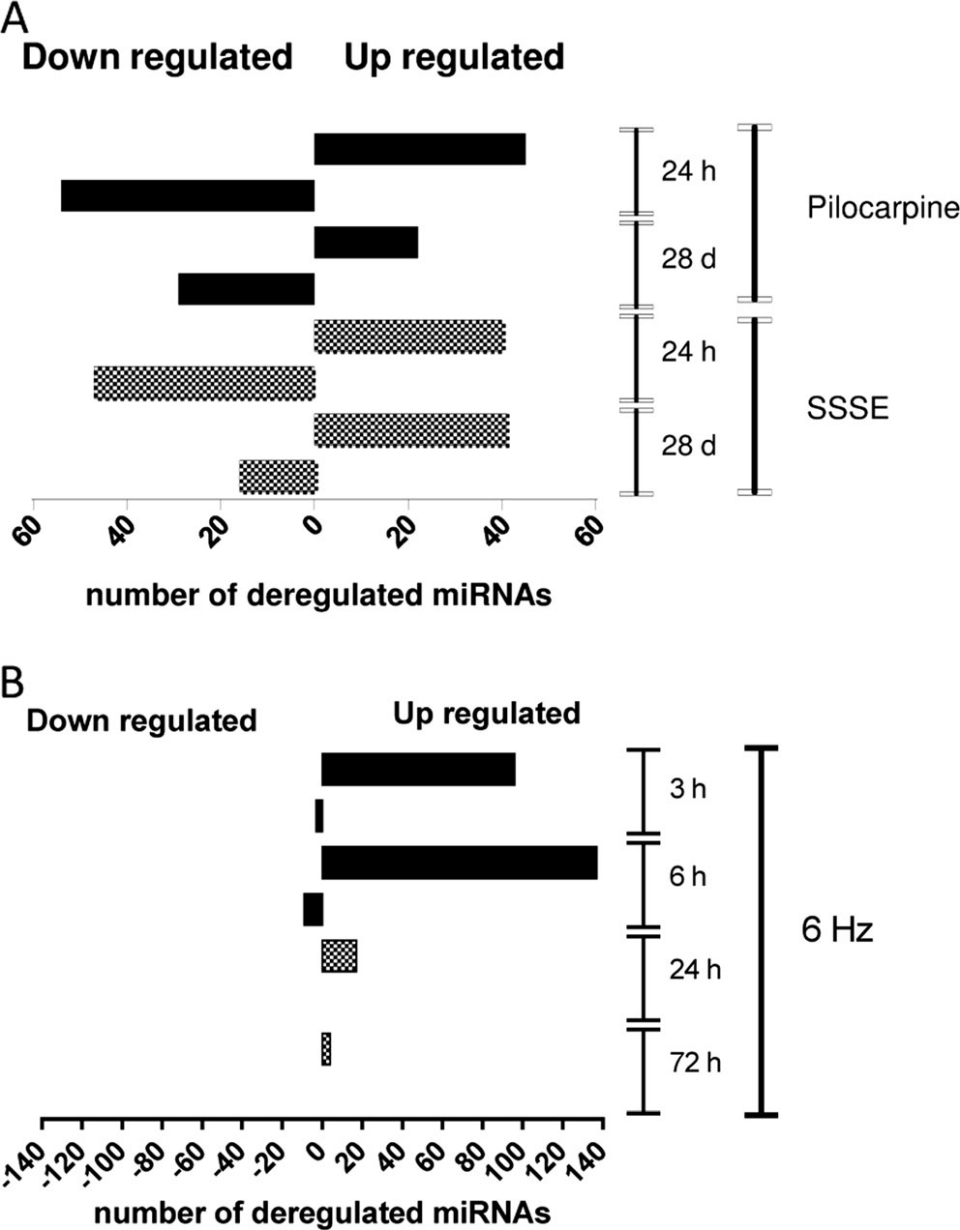
Differentially expressed miRNAs in the different mouse epilepsy models. Data represent the absolute number of up-regulated (*p* value >0.05 and FC > 1) or down-regulated (*p* value >0.05 and FC < 1) miRNAs for each time point and in each model. **a** Deregulated miRNAs in the pilocarpine and the SSSE model. **b** Deregulated miRNAs in the 6-Hz model

Comparison of individual time points within each model with their corresponding control groups revealed a number of differentially expressed miRNAs. All significantly changed miRNAs (Benjamini and Hochberg corrected *p* value <0.05) displaying a fold change (FC) of >1 were defined as up-regulated miRNAs, whereas miRNAs with a FC of <1 were defined as down-regulated (Fig. 6). The Supplementary Tables 2, 3 and 4 summarise the annotations of these differentially expressed miRNAs in all three models analysed.

In both chronic SE models, the total number of miRNAs with altered expression levels was higher at the early time point (24 h) relative to the later stage of the model (28 days). The absolute number of up- and down-regulated miRNAs was similar for both pilocarpine and SSSE animals (Fig. 6a). In the 6-Hz model, a higher proportion of miRNAs were up-regulated at early time points. Throughout all time points of the acute model, only very few miRNAs were down-regulated (three miRNAs were down-regulated at 3 and 8 h at 6 h and none at 24 and 72 h) (Fig. 6 and Supplementary Table 4). In addition, the total number of miRNAs with altered expression was much higher at 3 and 6 h compared with 24 and 72 h, respectively.

### Real-Time PCR Validation of Differentially Expressed miRNAs

**Fig. 7:**
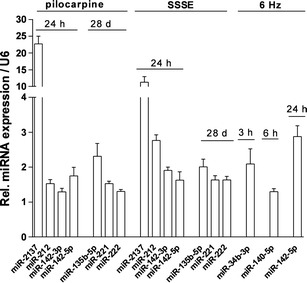
RT-qPCR validation of deregulated miRNAs. The expression of miRNAs which were identified as either up- or down-regulated 24 h and 28 days following SE in chronic epilepsy models and at 3, 6 and 24 h following seizure in acute epilepsy model was analysed by RT-qPCR. Data represent significant mean fold changes for *N* = 8 animals per group (*p* < 0.05 calculated by Student’s *t* test). RT-qPCR reactions that were performed in triplicates and fold changes were calculated by the ΔΔCt method using U6 small RNA as housekeeping gene and normalisation to control animal group. The controls were set as 1

To validate the differentially expressed miRNAs identified in our microarray analysis, we used qPCR and focused on commonly deregulated miRNAs among all three models as well as between the chronic models (Fig. 7). miRNA-142-5p commonly up-regulated at 24 h in all three models was successfully validated using qPCR. miRNA 331-3p and miRNA-30a-5p are both showing small fold changes in the microarray analysis and the expression levels in the qPCR were low, and no statistical difference was obtained using qPCR between the groups (data not shown). Next, we investigated miRNAs commonly deregulated in the two chronic epilepsy models 24 h following SE. miRNA-2137, miRNA-212 and miR-142-3p were significantly increased in comparison with the controls in both chronic epileptic models confirming the microarray data. The highest level of up-regulation was observed for miR-2137 at 24 h for pilocarpine and SSSE models (23-fold and 12-fold, respectively) (Fig. 7).

Furthermore, the qPCR analysis revealed that the expression of miRNA-221, miRNA-222 and miRNA-135b-5p was significantly increased in both chronic models at the 28-day time point (Fig. 7). In the 6-Hz model, miRNA-34b-3p was significantly up-regulated at 3 h, and miRNA-140-5p was significantly up-regulated at 6 h (Fig. 7). Overall, deregulation of selected miRNAs initially identified by microarray analysis was confirmed by qPCR.

### Pathway Analysis

In order to assess the impact of differentially expressed miRNAs on cellular mechanisms, an enrichment analysis was conducted for miRNA binding to genes belonging to all canonical pathway maps of the MetaCore™ software suite (http://thomsonreuters.com/site/systems-biology/). Only proven interactions with miRNAs recorded in MetaCore™ were considered.

When taking into account miRNAs down-regulated in both the pilocarpine and the SSSE models at 24 h post-SE, several pathways related to the mitochondrial metabolism were identified (data not shown). Table 1 shows the pathways linked with miRNAs up-regulated under the different experimental conditions. miRNAs up-regulated in samples from pilocarpine animals 24 h post-SE were enriched in several pathways associated with inflammation, such as IL-6, high-mobility group box 1 (HMGB1) and innate immunity. Despite several deregulated miRNAs, no strong correlation could be identified in the SSSE model relative to naïve animals. Nevertheless, miRNAs overexpressed in both pilocarpine and SSSE animal 24 h post-SE were slightly enriched in the same inflammation pathway even if not considered significant. In the acute seizure samples, up-regulated miRNAs after 3 and 6 h were enriched in several pathways involved in cell cycle regulation.

**Table 1:** Pathway analysis conducted with the MetaCore™ tool

Pathways	Pilocarpine		SSSE		6 Hz				Pilo vs SSSE		6 Hz
	24 h	28 days	24 h	28 days	3 h	6 h	24 h	72 h	24 h	28 days	3 vs 6 h
Neurophysiological process_GABAergic neurotransmission								0.038			
G-protein signalling_Rap1A regulation pathway						0.158		0.026			
Cell adhesion_Ephrin signalling						0.062		0.038			
Cannabinoid receptor signalling in nicotine addiction								0.038			
Cytoskeleton remodelling_Role of PKA in cytoskeleton reorganisation						0.042					
Cell cycle_Role of APC in cell cycle regulation					0.034						0.108
Apoptosis and survival_Lymphotoxin-beta receptor signalling	0.114				0.018	0.087					0.102
Cell cycle_Regulation of G1/S transition (part 2)					0.010	0.105					0.005
Cell cycle_Cell cycle (generic schema)					0.002	0.177					0.012
Benzo[a]pyrene metabolism					0.095	0.136					0.040
Neurophysiological process_Thyroliberin in cell hyper-polarisation and excitability					0.070	0.062					0.012
Blood coagulation_GPCRs in platelet aggregation					0.140	0.087					0.028
DNA damage_Inhibition of telomerase activity and cellular senescence					0.034	0.201					0.012
Development_Alpha-1 adrenergic receptors signalling via cAMP		0.001									
Development_Adiponectin signalling	0.032					0.234			0.254		
Immune response_Role of HMGB1 in dendritic cell maturation and migration	0.014								0.240		
Immune response_Role of PKR in stress-induced antiviral cell response	0.024					0.207					0.268
Mucin expression in CF via IL-6, IL-17 signalling pathways	0.028								0.185		
Mucin expression in CF via TLRs, EGFR signalling pathways	0.043								0.173		
Immune response_HMGB1 release from the cell	0.002								0.111		
Immune response_MIF in innate immunity response	0.042								0.173		

FDR values are shown for pilocarpine (pilo), SSSE and 6-Hz comparisons

*SSSE* self-sustained status epilepticus

## Discussion

miRNAs are important regulators of a plethora of pathophysiological processes, playing a prominent role in normal neuronal function as well as neurodegenerative diseases (Im and Kenny 2012; McNeill and Van Vactor 2012; Salta and De Strooper 2012). Several genome-wide miRNA screens provided evidence that miRNAs are differentially expressed in a variety of animal models of seizure (Aronica et al. 2010; Hu et al. 2012; Omran et al. 2012). However, available epilepsy animal models differ in several fundamental parameters such as induction of seizure/SE, time of seizure onset, frequency and severity of recurrent seizures, hippocampal sclerosis and microglia activation. These parameters most likely influence miRNA expression during different stages of epileptogenesis (Jimenez-Mateos and Henshall 2013). So far, to our knowledge, there is no comparative analysis of miRNA profiles in chronic models of TLE and acute seizure models. The identification of miRNAs related to spontaneous seizures in contrast to miRNAs deregulated in chronic stages of the disease is of essence for mechanistic understanding. miRNAs that are deregulated in the chronic phase are likely involved in fundamental processes of epileptogenesis and therefore interesting candidates for new therapeutic approaches. In order to detect miRNAs specific to disease progression and to distinguish them from miRNAs related to acute seizures only, we compared results from two chronic epilepsy models (pilocarpine and SSSE) with those from the acute 6-Hz seizure model. All three models used in our study share one important feature, namely the focal onset of seizures and clear involvement of temporal lobe structures (Barton et al. 2001; Mazzuferi et al. 2012). Therefore, miRNA profiling performed in the hippocampal samples obtained from these models should allow a good comparison of spatiotemporal expression changes.

Analysis of the results from three models revealed a clear separation in miRNA expression patterns in the acute single seizure model (6 Hz) and the two chronic epilepsy models (pilocarpine and SSSE). Thus, the induction of a single seizure induces a different miRNA expression patterns when compared to SRS following SE.

The high overlap of miRNAs at the early time point (24 h) for pilocarpine and SSSE models observed in the heat maps implies a certain similarity between both models, although they are based on different stimuli (chemical versus electrical) and were performed in different mouse strains (NMRI and C57Bl/6). A study by Parsons et al. (2008) showed that only ~5 % of 166 investigated miRNAs were differentially expressed in different mouse strains. Interestingly, some of the common miRNAs identified in our analysis at 24 h such as miR-27a and miR-146b are consistent with other post-SE mouse models (Jimenez-Mateos et al. 2011). Importantly, these miRNAs also show clear overlap with a rat TLE model (miR-146a, miR-135b, miR-27a, miR-210, etc.) (Hu et al. 2012). These observations strongly indicate common miRNA expression changes in response to SE, irrespective of the model, species or strain, suggesting their involvement in the epileptogenic process.

The 6-Hz single seizure model was designed to follow the alteration of miRNA expression over 72 h and to compare the expression pattern at 3, 6, 24 and 72 h with the starting point (0 h). Although in this experimental setting there are no individual controls for every time point, it allows for the analysis of the time-course of miRNA changes after a single seizure. The largest changes in this acute, single seizure model occur immediately following seizure (3 and 6 h). Alterations of miRNA expression return back to basal levels within 24 to 72 h in the 6-Hz model. This notion is further corroborated by the observation that 17 miRNAs were deregulated at 24 h and only 4 miRNAs were deregulated at 72 h.

For the overall comparison, the 24-h time point is of special interest, since it is common to all three models. Although 36 miRNAs were overlapping among the two chronic models, only three miRNAs overlapped between all models at 24 h. Among the three commonly regulated miRNAs, miR-142-5p is consistently up-regulated in all models. miR-142 is mainly associated with the immune system and plays a crucial role in myeloid (Chen et al. 2008; Wang et al. 2011) and macrophage differentiation, as well as IL-6 signalling (Sonda et al. 2013). Interestingly, our pathway analysis revealed a link between the identified miRNA pattern with genes associated with immune response in general and the IL-6 pathway in particular. Although the role of miR-142 in IL-6 regulation under epileptic conditions is not clear, it might be consistent with a role of this miRNA and IL-6 in epilepsy (Uludag et al. 2013) as well as neuroinflammation in general (Vezzani et al. 2012).

We focused on commonly deregulated miRNAs in chronic epilepsy models considering them as potentially relevant for the pathophysiological changes related to chronic forms of human epilepsy. The Venn diagrams show that 36 miRNAs were commonly altered at 24 h, whereas only 15 miRNAs were identified at 28 days. The majority of these miRNA candidates were confirmed by qPCR indicating a closer similarity in miRNA changes at early time points. Interestingly, among these miRNAs, miR-135b was previously described in the chronic stage of a rat TLE model (Hu et al. 2012). In addition, individual miRNAs at 28 days revealed a high overlap between our pilocarpine results and those from Hu et al. (Hu et al. 2012).

The observation that more miRNAs are commonly deregulated at 24 h than at 28 days is most probably due to different biological processes that are relevant for the specific time points (Riban et al. 2002). At 24 h, these include processes such as cell death, inflammation and gliosis, whereas at later stages of epileptogenesis, proliferation and sprouting of neurons are of importance (Riban et al. 2002).

The miRNA most consistently up-regulated in both chronic models was miR-2137. The qPCR analysis revealed a more than 12-fold increase of miR-2137 expression as compared with control in the SSSE model and a more than 23-fold increase in the pilocarpine model. Despite the increased expression of this miRNA in the acute phase of the SE models, very little is known about the biological implication of this miRNA in general. An additional complication in the analysis of miR-2137 is the absence of a human homolog.

Interestingly, we identified several miRNAs in the chronic phase of both models that have so far not been associated with seizures, for example miR-676, miR-467c/miR-467e, miR-191, miR-669c and miR-181. miR-181 regulates several cancer-associated genes (Neel and Lebrun 2013), but its role in astrocyte response to inflammatory stimuli (Hutchison et al. 2013) and regulation of GABA(A) receptors (Zhao et al. 2012) could be highly relevant for epilepsy.

miRNAs detected in our study have also been identified in other epilepsy models validating our experimental approach. For example, we confirmed the up-regulation of miR-146a, already described in other profiling studies in animal models and human epilepsy (Aronica et al. 2010; Hu et al. 2012; Omran et al. 2012). miRNA-146a has been described as being involved in astrocyte-mediated inflammatory response occurring 24 h after SE in the pilocarpine model (Mazzuferi et al. 2012) (RK personal communication). Interestingly, miR-146a is continuously up-regulated in the chronic phase of the SSSE model when astrocytosis takes place (RK personal communication). miR-21-3p and miR-21-5p are also commonly up-regulated in both post-SE models at 24 h. The expression of these miRNAs is modulated in many biological processes and in several pathological conditions and recently confirmed in children with mesial temporal lobe epilepsy (mTLE) (Peng et al. 2013). One possible mechanism linking these miRNAs with epilepsy could be the previously described targeting of the transcription factor myocyte enhancer factor 2C (MEF2C), which has been associated with neuronal dysfunction and neurodegeneration (Yelamanchili et al. 2010). Interestingly, mutations in MEF2C gene have been identified in patients with epilepsy (Paciorkowski et al. 2013). Moreover, miR-132 commonly up-regulated in the chronic models at 24 h for both models and at 28 days for pilocarpine has been identified to be increased in the CA3 region of animals 24 h post-SE. In addition, antagomiR-mediated depletion of miR-132 in animals reduced seizure-induced neuronal loss confirming the functional relevance of this miRNA in epilepsy (Jimenez-Mateos et al. 2011).

During the chronic phase of the post-SE models, we confirmed two miRNAs that have been shown to be associated with epilepsy in previous studies (Kan et al. 2012): miR-221 and miR-222. These two miRNAs were identified in profiling studies of animal epilepsy models or human TLE profiling studies (Kan et al. 2012) and shown to be down-regulated. In contrast, miR-221 and miR-222 were consistently up-regulated in our model. Both miRNAs are described to regulate the expression of intercellular adhesion molecule 1 (ICAM1, CD54) typically expressed on endothelial cells and cells of the immune system. This is consistent with the occurrence of neuroinflammation in a wide range of seizure syndromes (Vezzani et al. 2011, 2012) and the anticonvulsant and neuroprotective effect of anti-inflammatory treatment reducing microglial activation in post-SE models (Mazzuferi et al. 2013).

Individual miRNAs can target several mRNAs and thus affect entire pathways. A pathway analysis was performed on miRNAs deregulated in both chronic models and the acute 6-Hz model considering only proven miRNA-mRNA interactions as recorded in MetaCore™. Interestingly, this analysis revealed the regulation of several genes associated with inflammatory pathways such as IL-1β, IL-6 and HMGB1 in the early phase of the pilocarpine model. Surprisingly, we could not detect a similar association of inflammation with the early phase of the SSSE model but a slight enrichment when analysing the overlap of pilocarpine versus SSSE 24 h post-SE.

This prominent role of inflammatory pathways associated with seizures is consistent with observations from other groups describing active neuroinflammation in human epilepsy and animal seizure models. Among the variety of pro-inflammatory mediators, several cytokines have been studied in epilepsy. IL-1β exerts a pro-convulsive effect with worsened and prolonged seizure activity in rodent epilepsy models (Vezzani et al. 1999). Moreover, in brain tissue obtained from patients with TLE, IL-1β expression and IL-1 receptor are increased in astrocytes, microglia and neurons (Ravizza et al. 2008). For IL-6, pro-convulsive effects at high concentrations have been described in animal models even though IL-6 is necessary for the normal brain development (Lehtimaki et al. 2003). In humans, IL-6 increases significantly 24 h after generalised tonic-clonic seizures in the plasma and CSF (Peltola et al. 2000; Virta et al. 2002; Lehtimaki et al. 2007). Several other mechanisms including TNF-α and Toll-like receptor (TLR) signalling have been described in epilepsy (for review, see Balosso et al. 2013).

HMGB1 exerts different biological functions depending on its cellular localisation. HMGB1 can reach the extracellular space via two different routes: active secretion by stimulated cells and passive release from necrotic and apoptotic cells. HMBG1 is a member of the danger-associated molecular pattern (DAMPs) molecules and can activate several receptors including TLR4 and RAGE receptors that have pro-convulsive activity (Iori et al. 2013).

Interestingly, the pathway analysis also identified changes in cell cycle regulation after acute seizures in the 6-Hz model. This is consistent with previous observations that found neurogenesis and cell proliferation within the hippocampus in response to seizure induction (Jessberger et al. 2005; Siebzehnrubl and Blumcke 2008). Although the role of neurogenesis in epilepsy pathophysiology is still controversial, our results provide additional evidence for potential involvement of miRNAs in this process.

In conclusion, our comprehensive miRNA profiling study identified common miRNA signatures for the chronic epilepsy models with a clear distinction from the pattern observed in animals with a single seizure. The miRNAs that have also been found in other epilepsy-based miRNA profiling studies, together with newly identified miRNAs relevant to seizure disorders, should help improve our understanding of fundamental processes involved in epilepsy as well as processes leading to epileptogenesis. Our study could help elucidate novel mechanisms and therapeutic approaches in epilepsy.

## Electronic supplementary material

Below is the link to the electronic supplementary material.

Supplementary Figure 1 Outline and aim of the study. Expression pattern of miRNAs in three different epilepsy mouse models: pilocarpine, SSSE and 6 Hz models and their pattern of altered miRNAs were compared at different time points. In pilocarpine and SSSE model miRNAs from hippocampi were isolated at 24 h (serves as an early time point in chronic epilepsy model) and 28 d following SE (serves as a late time point in chronic epilepsy model). In the 6 Hz model miRNAs from hippocampi were isolated at 3 h, 6 h, 24 h and 72 h following acute seizure. Differentially expressed miRNAs obtained from miRNA microarrays were analyzed by PCA plots and shown as heat maps. Comparison of altered miRNAs pattern in different models was shown in Venn diagrams and validated by RT-qPCR. Most prominent miRNAs were analyzed in pathways prediction using the MetaCore™ tool. (PS 507 kb)

Supplementary Figure 2 Examples of EEG traces of spontaneous recurrent seizures occurring during the fourth week after pilocarpine-induced status epilepticus (panel A) or self-sustained status epilepticus (SSSE) (panel B). Pilocarpine model: a monopolar depth electrode was surgically implanted into the right hippocampal CA1 region (coordinates vs. bregma): AP = -1.94 mm, L = -1.0 mm, D = -1.25 mm. Three monopolar cortical electrodes, frontal left (AP = +1.0 mm, L = -2.0 mm lateral) and occipital left/right: AP = -4.0 mm, L = ±4 mm) were also positioned on the dura mater. A ground electrode was placed in the left prefrontal bone (Mazzuferi et al. 2012). SSSE model: were surgically implanted with EEG electrodes: depth electrode (bipolar) in the basolateral amygdala (coordinates vs. bregma): AP = -1.40 mm; L = -2.65 mm; D = -5.00 mm, cortical electrode (monopolar): AP = - 4.00 mm, L = + 3 mm and reference electrode in the prefrontal bone. (PS 578 kb)

Supplementary Figure 3 Weekly frequency (mean ± S.E.M.) of generalized seizures (stages 3-5 as described in methods section) displayed by mice in the pilocarpine and the self-sustained status epilepticus (SSSE) models within 4 weeks after induction of status epilepticus (see Material and Methods). The data was obtained by continuous video (in the pilocarpine model) or video-EEG monitoring (in the SSSE model). (PS 396 kb)

Supplementary Figure 4 Principle component analysis (PCA) based on all detected miRNAs. Scores of the samples in the first and the second principal components are shown. Clustering of samples of all three models (pilocarpine 24 h and 28 d, SSSE 24 h and 28 d and 6 Hz 0 h, 3 h, 6 h, 24 h and 72 h following seizure induction). (PS 4242 kb)

Supplementary Table 1 Overlapping miRNAs between pilocarpine and SSSE model at 24 h and 28 d. (XLSX 9 kb)

Supplementary Table 2 Most differentially regulated miRNAs in the Pilocarpine model at 24 h and 28 d following SE (p < 0.05). (XLSX 21 kb)

Supplementary Table 3 Most differentially regulated miRNAs in the SSSE model at 24 h and 28 d following SE (p < 0.05). (XLSX 21 kb)

Supplementary Table 4 Most differentially regulated miRNAs in the 6 Hz model at 3 h and 6 h following single seizure (p < 0.05). (XLSX 28 kb)
